# *Listeria monocytogenes* isolates from Western Cape, South Africa exhibit resistance to multiple antibiotics and contradicts certain global resistance patterns

**DOI:** 10.3934/microbiol.2021004

**Published:** 2021-01-19

**Authors:** Rochelle Keet, Diane Rip

**Affiliations:** Department of Food Science, Centre for Food Safety, Stellenbosch University, South Africa

**Keywords:** *Listeria monocytogenes*, listeriosis, antibiotic resistance, outbreaks, ready to eat foods, environment, South Africa

## Abstract

Food-borne disease outbreaks are common and offer valuable insights into the causes, impacts, and mechanisms underlying food pathogens. This also serves as a good foundation to validate the performance of current best practice control methods, for example antibiotics, that are used in the fight against food pathogens. Listeriosis outbreaks, caused by *Listeria monocytogenes*, is no exception. In 2018, South Africa experienced the largest global listeriosis outbreak recorded to date. However, despite the scale of this outbreak, information on the bacterium and its resistance towards antibiotics is still severely lacking. Furthermore, until now it remained to be determined whether *L. monocytogenes* antibiotic resistance patterns in South Africa mirror resistance patterns elsewhere in the world. The aim of this study was therefore to evaluate the efficacy of antibiotics that are currently used against *L. monocytogenes*. Using the European Committee on Antimicrobial Susceptibility Testing (EUCAST) disc diffusion method, *L. monocytogenes* isolates (n = 177) from diverse origins in the Western Cape, South Africa (clinical, food, and environment) were tested for susceptibility against five different antibiotics, namely ampicillin, erythromycin, chloramphenicol, gentamicin, and tetracycline. Isolates were collected over a period of two years (2017–2019). All isolates were susceptible to ampicillin, the currently recommended antibiotic, while a large number of isolates were resistant to chloramphenicol, erythromycin, and tetracycline. Also, patterns of resistance observed here are different to patterns observed elsewhere. The findings of this study demonstrate that it is imperative to continuously monitor the efficacy of currently recommended antibiotics, since resistance patterns can quickly develop when such antibiotics are overutilized, and secondly, that it is crucial to assess local antibiotic resistance patterns in conjunction with global patterns, since the latter is not necessarily generalizable to local scales.

## 1. Introduction

Foodborne illnesses, caused by food pathogens, are a major threat to public health the world over and are responsible for hundreds of thousands of human deaths every year [1]. One of the most important food pathogens is *Listeria monocytogenes*, a bacterium responsible for the serious, and often fatal, foodborne infection listeriosis [2]. It is ubiquitous in nature, occurring in soil, water, and fertilizer [3]. It is quite resilient, being able to withstand high levels of nitrite and salt, and can grow at very low temperatures (0–4 °C) [2],[4]. This means that *L. monocytogenes* can grow, and even thrive, in the refrigerator where other pathogens are often unable to grow [3],[5]. Because of the bacterium's characteristics, foods often implicated as a risk for carrying *L. monocytogenes* include ready-to-eat meat products (such as deli meat and sausages), cold-smoked fish, dairy products (especially soft cheeses), and fresh produce [2],[5]–[15]. In recent years, there have been several listeriosis outbreaks around the world associated with raw and ready-to-eat foods, so the presence of this pathogen in the food processing environment seems to be rising [16]–[19].

*L. monocytogenes* can further be classified into lineages I–IV [20],[21]. Despite lineage I isolates (1/2b, 4b) being overrepresented in human clinical cases, isolates from lineage II (serotypes 1/2a, 1/2c, and 3a) can also cause listeriosis [22]–[24]. Genetic mutations may enable isolates in certain lineage groups to either adapt to the food processing facility (due to environmental pressures) or to establish easier within hosts by crossing the intestinal barriers [22]. Isolates from a particular lineage carry different levels of risk and may associate with different origins.

Listeriosis can be especially detrimental in immunocompromised individuals, i.e., individuals with suboptimal immune systems such as pregnant women, neonates, the elderly, or patients with cancer, tuberculosis (TB) or human immunodeficiency virus (HIV) [2],[3],[25],[26]. The current preferred antibiotic treatment for listeriosis in these individuals is ampicillin, used as a standalone treatment or in combination with an aminoglycoside such as gentamicin [15],[27],[28]. For treating listeriosis in pregnant women, erythromycin is usually the antibiotic of choice [29]. Due to the high mortality rate of individuals that have contracted listeriosis, which can be as high as 30% among immunocompromised individuals, the emergence of antibiotic-resistant *L. monocytogenes* strains from food and the food processing environment is worrying (Table S1). Of particular concern, is the high reported resistance of *L. monocytogenes* (obtained from food sources) towards ampicillin and gentamicin [9],[30]–[32]. Fortunately, some antibiotics are still highly effective treatments against *L. monocytogenes* in clinical settings. For example, ampicillin and penicillin are still the most effective antibiotics against *L. monocytogenes*, with gentamicin being only slightly less effective [33]. In order for antibiotic treatment against *L. monocytogenes* to be effective, the antibiotics should be able to penetrate and distribute within the host cell [4]. Besides the general use as treatment of infectious diseases, antibiotics are also used in the prevention of disease and growth promotion in the animal production industry (e.g., tetracycline), as well as biocides in household and toiletry products (e.g. triclosan) [34]–[37]. Different geographical environments, as well as the different applications of antibiotics (e.g. clinical or veterinary use), can have an influence on the resistance or susceptibility patterns of *L. monocytogenes* isolates [8],[38].

In the past few years, a steady increase in the presence of *L. monocytogenes* in various food types have led to a number of listeriosis outbreaks around the world [17]–[19],[39]. This together with an associated mortality rate of as high as 30%, means the potential antibiotic resistance of *L. monocytogenes* is a rising and important public health concern.

In South Africa, a listeriosis outbreak in 2017–18 was thought to be the largest global outbreak on record, with 1 060 cases and 216 deaths reported (up to 20.4% mortality) [40]. Here, listerial meningitis is currently the second most common cause of bacterial meningitis [41]. In the past, patients that presented with meningitis symptoms were treated with ampicillin and gentamicin. Cephalosporins are commonly used to treat non-listerial meningitis, since *L. monocytogenes* are intrinsically resistant to this class of antibiotics. With the emergence of the 2017–18 outbreak, patients in South Africa showing signs of meningitis were treated with third-generation cephalosporins, as well as a course of ampicillin with gentamicin [27],[42].

Currently, there is a general paucity of information in South African literature on the antibiotic resistance patterns of *L. monocytogenes* isolates from the food and clinical environment. Prior to the outbreak, listeriosis was a non-notifiable disease, i.e. health workers were not responsible for informing the health authorities when dealing with cases of listeriosis [43],[44]. Therefore, the same amount and quality of historic data that other countries (e.g. the United States) possess regarding listeriosis outbreaks and cases in general is not available in South Africa. This, together with the high levels of mortality associated with the recent outbreak and the fact that antibiotic-resistant strains of *L. monocytogenes* are generally on the rise, warranted an investigation into the efficacy of antibiotics used against *L. monocytogenes*. The aim of this study was thus to assess the antibiotic susceptibility patterns of *L. monocytogenes* isolates from clinical, food, and environmental origin, collected over a period of two years (2017–2019) in the Western Cape, South Africa. The first objective was to determine the efficacy of various different antibiotics that are currently prescribed for use against *L. monocytogenes*. The second objective was to determine whether foods of different origins/categories differ in their patterns of resistance. Finally, the third objective was to identify whether current resistance patterns in South Africa are in line with global resistance patterns or not.

## 2. Materials and methods

### 2.1. Sample acquisitions

A total of 177 *Listeria monocytogenes* isolates were collected from various origins: clinical, environmental, raw seafood, raw meats, and ready-to-eat foods (Table 1). Clinical isolates were obtained on 2% blood agar from the National Health Laboratory Services (NHLS) (bacterial isolates did not have patient identifiers and no contact with any human specimens were made), while all other isolates were obtained from a food accredited laboratory (Microchem Lab Services, Cape Town) on RAPID'L.mono plates (AEC Amersham). All isolates were further subdivided into subcategories within their respective categories, where enough information was provided (for example subdividing Environmental samples into equipment, drain, surfaces, etc.) (Table 1). For the remainder of the article, ‘Ready-to-eat’ refers specifically to one of the main categories in this study, whereas ‘RTE’ refers to ready-to-eat foods in general. See supplementary information (Table S2) for a more detailed description on sample acquisition. This study was approved by the Research Ethics Committee: Biosafety and Environmental Ethics (REC:BEE), Stellenbosch University (BEE-2018-1764) and Faculty of Health Sciences, Human Research Ethics Committee, University of Cape Town (HREC R020/2015).

**Table 1.** Various categories and subcategories of products that were tested.

Clinical	Environmental	Raw (meat and chicken)	Raw seafood	Ready-to-eat
Clinical	Drain	Beef	Seafood	Dairy
	Equipment	Chicken		Deli meat
	Floor	Pork		Fresh produce
	Hand	Fresh produce		Hummus
	Surface			Polony

### 2.2. DNA extractions and genotyping

Presumptive positive colonies (blue-black) from RAPID'L.mono plates (AEC Amersham) were sub-cultured on 2% blood agar (NHLS, Greenpoint) for overnight growth at 37 °C. All cells recovered from blood agar were subjected to DNA extraction using the Quick-DNA™ Fungal/Bacterial Miniprep Kit (ZymoResearch) according to the manufacturer's instructions. Strains were confirmed as *L. monocytogenes* by using polymerase chain reaction (PCR) to amplify a 730 bp region of the *hly*A gene [45],[46], which is innate to all *L. monocytogenes* bacteria and encodes for a pore-forming haemolysin [47]–[50]. For added lineage characterisation, isolates were characterised as either lineage I, II or III by a SNP based PCR-RFLP method designed by an author of this study [51]. Briefly, the *hly*A amplicons underwent restriction digestion with enzymes *Nde*I, *Hae*II and *Bsh*1285I to produce band sizes indicative of lineage I, II or III respectively [51]. All amplicons and restriction digests were visualised by gel electrophoresis on a 1.5% agarose gel (Lonza, WhiteHead Scientific), stained with SmartGlowTM pre-stain (Whitehead Scientific).

### 2.3. Antibiotic susceptibility tests

All isolates that were included for antibiotic susceptibility testing, had tested positive for the 730 bp *hly*A gene fragment with added lineage group information.

*Listeria monocytogenes* isolates stored as glycerol stocks were streaked onto tryptic soy agar (TSA) (Oxoid™, ThermoFischer Scientific) and grown overnight at 37 °C. Antibiotic susceptibility testing was performed by a disc diffusion method according to guidelines by the European Committee on Antimicrobial Susceptibility Testing [52]. Briefly, overnight cultures of *L. monocytogenes* were suspended in 0.85% saline solution (Oxoid™, ThermoFischer Scientific) to obtain a McFarland standard of 0.5 using the McFarland equivalence turbidity standard (Oxoid™, ThermoFischer Scientific). Subsequently, it was swabbed (using cotton tip swabs) onto Mueller-Hinton agar supplemented with 5% defibrinated horse blood and 20 mg/L β-NAD (MH-F agar) (Oxoid™, ThermoFischer Scientific). Antibiotic discs namely ampicillin, erythromycin, chloramphenicol, gentamicin, and tetracycline (Oxoid™, ThermoFischer Scientific), were applied using sterile forceps and agar plates were incubated for 20–24 h at 37 °C, after which the zones of inhibition were measured (Table 2). *Staphylococcus aureus* ATCC 25923 (Davies Diagnostics) was used as a control strain [53]–[55]. The zones of inhibition were classified as susceptible or resistant [52]. Isolates resistant to at least one antibiotic from three or more different antimicrobial categories were classified as multi-drug resistant (MDR) [56]. The choice of antibiotics tested was based on its clinical relevance, as well as the emergence of resistance against these antibiotics in the environment [9],[29].

**Table 2.** Isolates were classified as susceptible (S) or resistant (R) based on the zones of inhibition (mm) observed; isolates that were resistant to at least one antimicrobial from three or more antimicrobial categories were classified as MDR [52].

Antibiotic	Antimicrobial category	Mode of action	Disc content (µg)	Zone diameter breakpoint (mm) [52]	
				S	R
Ampicillin	Beta-lactams	Bacterial cell wall synthesis inhibitor	10	≥16	<16
Chloramphenicol*	Phenicols	Inhibits protein synthesis (prevents growth)	30	≥18	<18
Erythromycin	Macrolides	Inhibits protein synthesis (bacteriostatic)	15	≥25	<25
Gentamicin*	Aminoglycoside	Inhibits protein synthesis (leads to cell death)	10	≥18	<18
Tetracycline*	Tetracycline	Inhibits protein synthesis (ribosomal inhibitor)	30	≥22	<19

*Breakpoints of *Staphylococcus aureus* ATCC 25923 were used to interpret the zones of inhibition [53],[57],[58].

### 2.4. Statistical analysis

All statistical analyses were conducted in the R statistical environment (version 3.5.1) [59]. In order to determine whether or not antibiotic resistance patterns were statistically significant, binomial tests were conducted separately for each category. Tests were also performed separately for each antibiotic. Binomial tests were chosen since experimental outcomes were all binary, i.e. isolates were either classified as susceptible or resistant according to the aforementioned methods. Expected probabilities of 0.5 were used in all instances, in order to test the hypothesis that antibiotic effectiveness was equal to 50% (i.e., no better than random chance). The binomial tests were performed with the function binom.test from the base package.

## 3. Results

**Figure 1.**
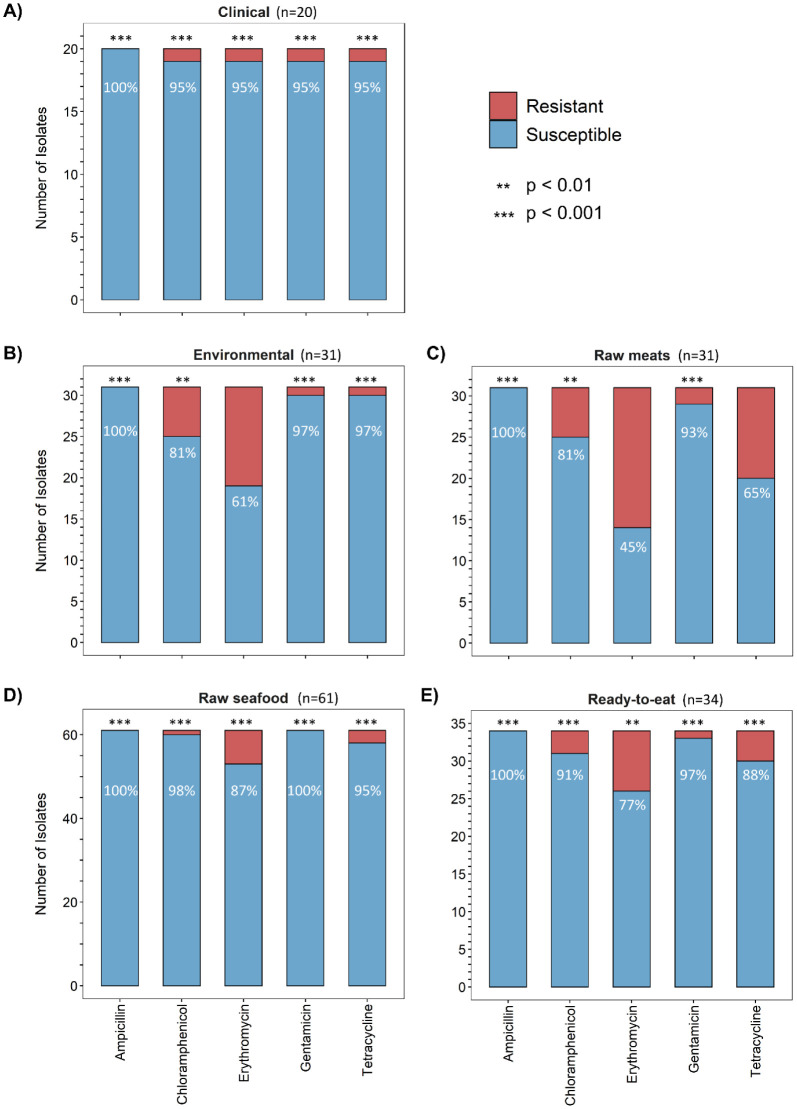
Susceptibility of *Listeria monocytogenes* isolates from main categories to respective antibiotics: Clinical (A), Environmental (B), Raw meats (C), Raw seafood (D), and Ready-to-eat (E).

Antibiotic susceptibility tests indicated that all the isolates (n = 177) were susceptible to ampicillin. Isolates from the Clinical category (n = 20) were susceptible (p < 0.001) to all five antibiotics tested (Figure 1A). Interestingly, one MDR isolate from the Clinical category showed resistance to four antibiotics, namely chloramphenicol, erythromycin, gentamicin, and tetracycline.

Isolates from Raw seafood category (n = 61) were susceptible (p < 0.001) to all five antibiotics tested (Figure 1D). There were a few isolates from this category resistant to chloramphenicol (n = 1), erythromycin (n = 8), and tetracycline (n = 3), which is in agreement with other studies conducted on *L. monocytogenes* isolated from seafood [9],[60]. No MDR isolates were detected in this category.

**Figure 2.**
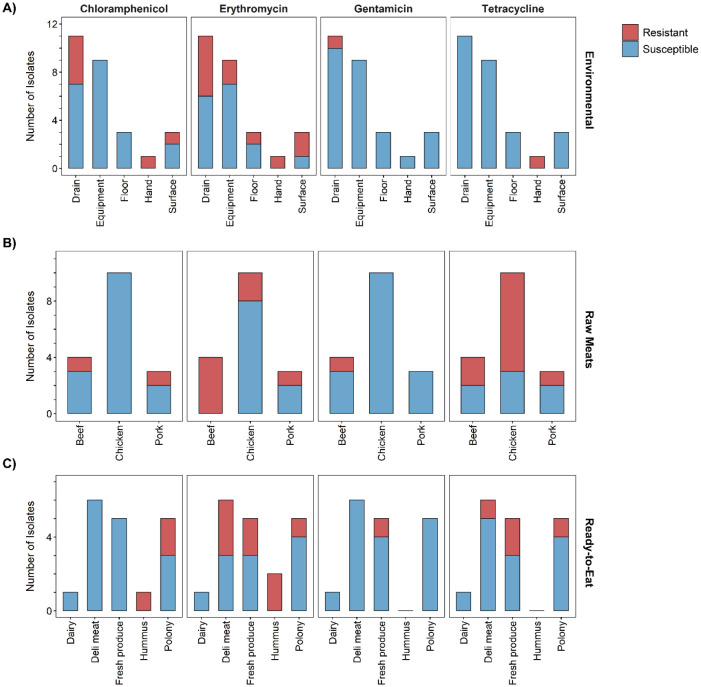
Susceptibility of *Listeria monocytogenes* isolates from subcategories Environmental (A), Raw meats (B), and Ready-to-eat (C) to respective antibiotics.

Isolates from the Ready-to-eat category (n = 34) were susceptible (p < 0.001) to all antibiotics tested (Figure 1E). Further subdivision of the Ready-to-eat category revealed that hummus isolates (n = 15) were all susceptible to gentamicin and tetracycline (Figure 2C), however, there were isolates resistant to chloramphenicol (n = 1) and erythromycin (n = 2). Isolates from deli meat (n = 6) were all susceptible to chloramphenicol and gentamicin, but there were isolates resistant to erythromycin (n = 3) and tetracycline (n = 1). Isolates originating from polony (processed meat product similar to bologna sausage) (n = 5) were all susceptible to gentamicin, however there were isolates that showed resistance to chloramphenicol (n = 2), erythromycin (n = 1), and tetracycline (n = 1). One isolate from this category was MDR. Chloramphenicol- and tetracycline resistance have been reported previously among *L. monocytogenes* isolates from RTE meat products [31],[54]. The fresh produce isolates (n = 5) were all susceptible to chloramphenicol, however there were isolates displaying resistance to erythromycin (n = 2), gentamicin (n = 1) and tetracycline (n = 2). One of these isolates (originating from coriander) was resistant to all three of the antibiotics and therefore was classed as MDR. The one dairy isolate was susceptible to all antibiotics.

In the Environmental category (Figure 1B), isolates were susceptible to ampicillin, chloramphenicol (p < 0.01), gentamicin and tetracycline (both p < 0.001). A smaller fraction of Environmental isolates (n = 31) were resistant to erythromycin (n = 12).

Further subdivision of the Environmental category (n = 31) (Figure 2A) indicated that the isolates originating from food processing equipment (n = 9) were all susceptible to chloramphenicol, gentamicin, and tetracycline. However, there were two isolates resistant to erythromycin. The isolates from factory drains (n = 11) were all susceptible to tetracycline, while being resistant to gentamicin (n = 1), chloramphenicol (n = 4) and erythromycin (n = 5). The MDR isolate from a food-factory worker's hand i.e ‘hand’ isolate (n = 1) was resistant to chloramphenicol, erythromycin, and tetracycline. The food contact surface i.e. ‘surface’ isolates (n = 3) were all susceptible to gentamicin and tetracycline, while a greater fraction of these samples were resistant to erythromycin (n = 2). One isolate from floor was resistant to erythromycin.

The Raw meats isolates (n = 31) (Figure 1C) showed susceptibility to ampicillin (p < 0.001), chloramphenicol (p < 0.01) and gentamicin (p < 0.001), with a smaller fraction of isolates showing resistance to tetracycline (n = 11). Slightly more than half of the isolates showed resistance to erythromycin (n = 17). Isolates from this category (n = 31) also exhibited the highest level of multidrug resistance (n = 4).

Subdivision of the Raw meats category (n = 31) (Figure 2B) revealed that the chicken isolates (n = 10) were all susceptible to chloramphenicol and gentamicin. While there were a few isolates resistant to erythromycin (n = 2), a large fraction was resistant to tetracycline (n = 7). All the beef isolates (n = 4) were resistant to erythromycin, with some showing resistance to chloramphenicol (n = 1), gentamicin (n = 1), and tetracycline (n = 2). In this category, two MDR beef isolates were observed (Table S2). Pork isolates (n = 3) were all susceptible to gentamicin, while one of the isolates in this category showed multidrug resistance to chloramphenicol, erythromycin and tetracycline. Four MDR strains were reported in the Raw meats category (Table S2).

Observing statistical significance for antibiotic susceptibility in the main categories of origin was reassuring, since it indicates that antibiotics are performing better than random chance. However, it should be noted that a single MDR strain, resistant to antibiotics used in human medicine, is a cause for concern, since such a strain could eventually transfer resistance to other strains, and could itself establish and proliferate. Thus, antibiotic resistant bacteria are, without exception, unwanted in any setting at any time and their existence is therefore never trivial, irrespective of the amount of strains found.

**Figure 3.**
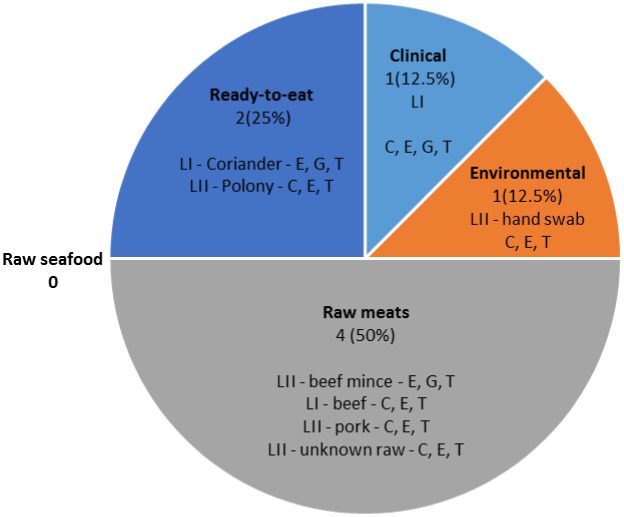
MDR isolates (n = 8) were mostly observed in samples originating from Raw meats, while the rest originated from Ready-to-eat, Clinical and Environmental. Resistance to E (erythromycin), G (gentamicin), T (tetracycline), and C (chloramphenicol) are shown, as well as their respective classification into LI (lineage I) and LII (lineage II).

The eight isolates that exhibited multidrug resistance, were all resistant to erythromycin and tetracycline. The eight MDR isolates (Figure 3) included: Raw meats (n = 4), Ready-to-eat (n = 2), Environmental (n = 1) and Clinical (n = 1). There were no MDR isolates in the Raw seafood category. Six of the eight MDR isolates were resistant to chloramphenicol, erythromycin and tetracycline.

Molecular characterisation by PCR-RFLPs revealed a spread of Lineage I and II isolates across food processing facilities and food categories (Table S2).

## 4. Discussion

The fact that none of the isolates were resistant to ampicillin was encouraging, as this is the current antibiotic of choice against *L. monocytogenes* in South African clinics and hospitals [27],[41]. As with our study, other researchers have also reported 100% susceptibility to ampicillin [61]–[63]. However in stark contrast to this, a number of international studies have recently demonstrated a high presence of ampicillin resistant strains in raw meats [30],[64]–[66], dairy [32], RTE foods [31],[53], seafood [9],[60], and clinical isolates [67]. Thus, while in South Africa ampicillin is still regarded as an effective treatment against *L. monocytogenes*, the aforementioned studies indicate previously effective antibiotics can lose their efficacy. The situation in South Africa should, therefore, be carefully monitored since resistant strains of *L. monocytogenes* might arise or be introduced from elsewhere, which could have devastating consequences.

### 4.1. Clinical isolates show a high level of susceptibility

One Clinical isolate was resistant to four antibiotics, namely chloramphenicol, erythromycin, gentamicin, and tetracycline. Resistance to these antibiotics among clinical strains have been reported [64],[67]. Noll *et al*. [57] also reported the occurrence of multidrug resistance among strains from human listeriosis cases (56%, n = 259), obtained during an outbreak in Austria and Germany, but found no resistance against the two antibiotics of choice, namely ampicillin and gentamicin. While it is encouraging that the majority of Clinical isolates were susceptible, the presence of a MDR strain is concerning.

In South Africa, antibiotic resistant bacteria amongst other diseases (such as TB) is on the rise [68]. We showed that antibiotic resistance was much more prevalent in environmental and food categories as compared to clinical. Other studies have likewise demonstrated a higher prevalence of *L. monocytogenes* resistance among environmental isolates compared to clinical [56],[67],[69]–[72]. Reasons for bacteria acquiring resistance include: medical patients neglecting to complete the full antibiotics courses [73] and long-term exposure to triclosan (found in many household products), which at sub-lethal levels, could potentially increase resistance in non-pathogenic bacteria. This could in turn transfer aminoglycoside (e.g. gentamicin) resistance, via horizontal gene transfer, to *L. monocytogenes* [35],[36],[38],[74].

### 4.2. Multidrug resistance among *Listeria monocytogenes* isolates

MDR bacteria are especially problematic since they are associated with a higher mortality rate than susceptible bacteria, and also result in higher cost of hospitalization as antibiotic therapy often needs to be extended [75]–[77]. MDR strains circulating in the environment can be transferred to food processing plants via incoming raw material or factory personnel. It is concerning that the isolate obtained from a factory worker's hand was resistant to three different antibiotics (Figure 3). This is a potential threat to consumers, should it spread to food consumed without further cooking. By introducing MDR strains into a processing facility, strains could circulate and spread in the immediate environment. Non-pathogenic MDR bacteria might not be problematic *per se*, however horizontal gene transfer could lead pathogenic bacteria to acquire resistance genes from non-pathogenic bacteria [35],[63]. Soil studies have revealed that high amounts of antibiotic compounds have accumulated in these environments over time, selectively pressurizing non-pathogenic soil bacteria to adapt and develop resistance genes (‘resistome’) [78].

Additionally, it is also known that *L. monocytogenes* can acquire erythromycin and tetracycline resistance from lactic acid bacteria, but this transfer is however influenced by the food medium [38].

There were eight MDR isolates identified (Figure 3); lineage I (n = 3) and lineage II (n = 5). All eight isolates were resistant to erythromycin and tetracycline whilst six were resistant to erythromycin, tetracycline and chloramphenicol (lineage I (n = 2), lineage II (n = 4)). Three MDR isolates in different categories showed resistance to gentamicin (lineage I, n = 2). Only two other non-MDR isolates in this study were resistant to gentamicin, both of lineage I origin (Table S2). From the MDR isolates found, there was no predisposition of isolates from one lineage group to resistance to a specific antibiotic, highlighting that MDR isolates within different lineages could disseminate in the environment and pose a health threat.

### 4.3. The use of antibiotics and disinfectants at sub-inhibitory levels influence antibiotic resistance

Similar to ours, previous studies of *L. monocytogenes* isolates originating from poultry, pork, and other meats, exhibited resistance to erythromycin and tetracycline [30],[65],[79]. However, our MDR isolates from this category also exhibited resistance to gentamicin and chloramphenicol. Tetracycline resistance is the most frequently reported antibiotic resistance among *L. monocytogenes* species [8],[69],[71],[80]. Our study aligns with this observation since levels of tetracycline resistance were high among the raw meat isolates tested here. This is not surprising since antibiotics are often used in sub-therapeutic levels in the poultry and meat industries, leading to reduced antibiotic effectiveness [30],[38],[81]–[86]. Furthermore, tetracycline-resistant strains of *L. monocytogenes* have already been isolated from the meat processing environment [85], as well as raw meat products. In South Africa, tetracycline contributes to 17% of the total amount of antibiotics used in the agricultural industry [87]. There have been several reports outside of South Africa of accumulated tetracycline residues detected in chicken meat [37],[88]–[90]. As of 2006, all classes of antibiotics (used as growth promoters) have been banned in the European Union [91], but in many developing countries (including South Africa), these are still being used in agriculture. This could explain the high number of Environmental and Raw meats isolates resistant to erythromycin, tetracycline, and chloramphenicol, in comparison to findings in other countries.

Antibiotic resistance also occurred among *L. monocytogenes* isolates from fresh produce (e.g. coriander) [92],[93]. Isolates in our study were resistant to erythromycin, gentamicin, and tetracycline. Similarly, erythromycin and tetracycline resistant *L. monocytogenes* have been previously isolated from vegetables [92],[93]. Aminoglycosides (which include gentamicin) are used in plant crop industries to control fire blight [94]. The sub-therapeutic use of these antibiotics is highly concerning since some of these antibiotics are not completely broken down during cooking processes, leaving residues behind that are ingested by the consumer [95]. This can lead to a disturbance in the consumer's microflora, due to continuous exposure to small amounts of antibiotic residues [84],[94]. RTE foods do not undergo heat treatment prior to consumption, meaning that individuals are at greater risk for listeriosis if they consume these types of foods. The occurrence of a MDR *L. monocytogenes* in coriander (this study) highlights the importance of washing any fresh produce prior to consumption.

In addition to antibiotic use, biocides and disinfectants are also used in the processing of raw meat. It has been suggested that the sub-inhibitory use of these decontaminants may lead to bacteria acquiring antibiotic resistance [61],[96]. It is also believed that exposure of *L. monocytogenes* to stressful conditions (e.g. osmotic stress, temperature fluctuations etc.) within the food processing environment can have an effect on its resistance to clinical antimicrobials [38]. This could explain why a higher number of environmental *L. monocytogenes* isolates showed resistance to several antibiotics. Furthermore, exposure to sub-inhibitory concentrations of poultry-washing chemicals leads *L. monocytogenes* and *Salmonella enterica* to acquire resistance to multiple antibiotics, including erythromycin and chloramphenicol [61]. This is consistent with our results, namely: *L. monocytogenes* exhibited resistance to multiple antibiotics, specifically erythromycin, chloramphenicol, and tetracycline. Although few studies have attempted to investigate the correlation between decontamination procedures and possible antibiotic resistance, the high levels of *L. monocytogenes* resistance found in raw meat and chicken samples in our study suggests that such a correlation potentially exists. Because resistant bacteria are already present in the environment, the continued sub-therapeutic use of antibiotics will put even more selective pressure on these bacteria, which will lead to an increase of resistance genes [35],[74]. Thus, current regulations should be scrutinized and updated so that the effects of such therapeutic use can be minimized.

### 4.4. *Listeria monocytogenes* antibiotic resistance patterns are geographically biased

**Figure 4.**
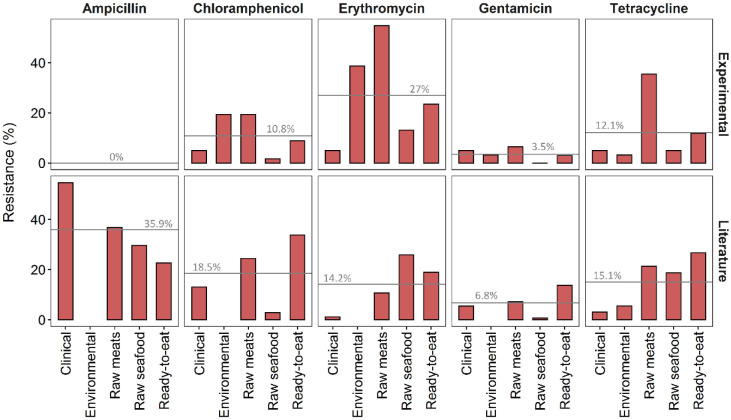
Antibiotic resistant *L. monocytogenes* comparison between experimental (n = 177 isolates in this study) and literature (n = 48 studies; Table S1); grey lines indicate mean values of resistance across all categories for each antibiotic.

Patterns of *L. monocytogenes* antibiotic resistance seem to be geographically biased in that they differ across various regions of origin (Figure 4). When comparing results of other researchers with the results from this study, different patterns of antibiotic resistance emerged, especially for ampicillin and erythromycin. While the exact drivers and mechanisms underlying such differences are still unclear, additional studies on the resistance of *L. monocytogenes* isolates from the South African environment would be of great benefit to determine firstly whether the patterns in this study remain consistent if replicated in a similar fashion, and secondly why these patterns differ so greatly from other countries. Finally, an investigation into whether differences in antibiotic use influences resistance patterns in different countries would be invaluable.

## 5. Conclusion

To the best of our knowledge, this is the first study in South Africa in which antibiotic resistance for *L. monocytogenes* was determined simultaneously for isolates from both clinical and various food origins. We determined that ampicillin was still the most effective treatment against *L. monocytogenes* irrespective of origin. We also showed that isolates from all categories were resistant to chloramphenicol, erythromycin, and tetracycline, with a large number of isolates showing resistance to these antibiotics. Erythromycin is the preferred antibiotic for treating listeriosis in pregnant women [29]. From a clinical perspective, the high resistance to erythromycin in isolates from this study, is of particular concern. Because erythromycin is commonly used a growth promoter in the agricultural industry, it could explain the results we obtained [84],[91]. From literature, there is a definite correlation between the use of antibiotics in the agricultural and clinical industry and the appearance of antibiotic resistance [74]. Information on the agricultural use of antibiotics in South Africa is scarce, with only a handful of studies attempting to quantify this amount [91],[97].

In a country such as South Africa that has a high burden of diseases such as TB and HIV/AIDS, the increase in antibiotic resistant pathogenic *L. monocytogenes* strains could increase the number of annual fatalities of such immunocompromised individuals [68],[82],[98]. Therefore, it is vital to continue surveillance studies tracking antibiotic resistant *L. monocytogenes* strains. Understanding the degree of antibiotic resistance among bacteria could lead to better administration of antibiotics, and in the case of *L. monocytogenes,* could help reduce mortality rates from listeriosis [68]. We observed that antibiotic resistance patterns differ from country to country [30],[99]. Thus, antibiotic resistance patterns in South Africa might not be relatable to that which is reported in other countries. Finally, eight MDR isolates were observed from various origins and categories (RTE, clinical, environmental, raw meats), and such strains specifically are of great concern due to their potential impact on human health, especially if such strains become dominant in the food processing environment. Three MDR isolates (obtained from raw beef, coriander, and clinical) were resistant to gentamicin. This is especially concerning as gentamicin is one of the antibiotics of choice in South Africa.

This work also provides insight to the food industry and health sector on the type of antibiotic resistant *L. monocytogenes* strains circulating in the South African environment.
