# Supplementary material for: Listeria monocytogenes isolates from Western Cape, South Africa exhibit resistance to multiple antibiotics and contradicts certain global resistance patterns
Source: AIMS Microbiol. 2021 Jan 19;7(1):40–58. doi: 10.3934/microbiol.2021004 (PMC7921373; doi:10.3934/microbiol.2021004)
Supplement: Supplementary file 1 [file microbiol-07-01-004-s001.pdf]

AIMS Microbiology

AIMS Microbiology, 7(1): 40–58.
DOI: 10.3934/microbiol.2021004
Received: 26 October 2020
Accepted: 04 January 2021
Published: 19 January 2021

http://www.aimspress.com/journal/microbiology

Research article

Listeria monocytogenes isolates from Western Cape, South Africa exhibit resistance to multiple antibiotics and contradicts certain global resistance patterns

Rochelle Keet and Diane Rip*

Department of Food Science, Centre for Food Safety, Stellenbosch University, South Africa

* **Correspondence:** Email: dirip@sun.ac.za.

Table S1. Studies indicating the prevalence of antibiotic resistance of *Listeria monocytogenes* to ampicillin, chloramphenicol, erythromycin, gentamicin, and tetracycline

Antibiotic	Origin	Reference	*L. monocytogenes* isolates resistant/isolates tested (%)
Ampicillin	Clinical, USA	[67]	6/65 (9.2%)
	Clinical, Iran	[64]	14/14 (100%)
	Seafood, Iran	[9]	107/278 (38.5%)
	Seafood (open-air fish markets), Iran	[60]	9/43 (20.9%)
	Chicken, Iran	[64]	14/14 (100%)
	Poultry, Iran	[30]	44/98 (44.9%)
	Raw meat, Italy	[65]	8/40 (20%)
	Pork, China	[79]	3/26 (11.5%)
	Meat products, Turkey	[66]	5/66 (7.5%)
	Dairy products, Iran	[32]	5/19 (26.3%)
	RTE products, Spain	[31]	8/25 (32%)
	RTE products, Poland	[53]	20/210 (9.5%)

Continued on next page

Antibiotic	Origin	Reference	*L. monocytogenes* isolates resistant/isolates tested (%)
Chloramphenicol	Clinical, USA	[67]	7/54 (12.9%)
	Seafood, Iran	[9]	9/278 (3.2%)
	Seafood (open-air fish markets), Iran	[60]	1/43 (2.3%)
	Poultry, Iran	[30]	24/98 (24.5%)
	Dairy products, Iran	[32]	2/19 (10.5%)
	Vegetables, Nigeria	[93]	60/104 (57.7%)
	RTE meat products, China	[100]	11/33 (33.3%)
Erythromycin	Clinical, USA	[67]	1/84 (1.9%)
	Seafood, Iran	[9]	7/278 (2.5%)
	Seafood (open-air fish markets), Iran	[60]	12/43 (27.9%)
	Seafood, Poland	[101]	33/70 (47.1%)
	Poultry, Iran	[30]	15/98 (15.3%)
	Raw meat, Italy	[65]	2/40 (5%)
	Pork, China	[79]	3/26 (11.5%)
	Dairy products, Iran	[32]	3/19 (15.8%)
	Vegetables, Nigeria	[93]	23/104 (22.1%)
Gentamycin	Clinical, USA	[67]	2/52 (2%)
	Clinical, Iran	[64]	1/14 (7.1%)
	Seafood, Iran	[9]	2/278 (0.72%)
	Chicken, Iran	[64]	1/14 (7.1%)
	Poultry, Iran	[30]	10/98 (10.2%)
	Raw meat, Italy	[65]	3/40 (7.5%)
	Pork, China	[79]	1/26 (3.9%)
	Dairy products, Iran	[32]	1/19 (5.3%)
	Vegetables, Nigeria	[93]	33/104 (31.7%)
	RTE products, Spain	[31]	1/25 (4%)
Tetracycline	Clinical, USA	[67]	2/66 (3%)
	Seafood, Iran	[9]	52/278 (18.7%)
	Poultry, Iran	[30]	34/98 (34.7%)
	Raw meat, Italy	[65]	1/40 (2.5%)
	Chilled pork, China	[79]	7/26 (26.9%)
	Dairy products, Iran	[32]	3/19 (15.8%)
	Vegetables, Nigeria	[93]	32/104 (30.8%)
	RTE products, Spain	[31]	12/25 (48%)
	RTE meat products, China	[100]	4/33 (12.1%)
	Food processing environment, Canada	[102]	3/54 (5.6%)

Table S2. *Listeria monocytogenes* isolates from the period 2017–2019 showing resistance to one or more antibiotic in various categories (isolates susceptible to all antibiotics not shown). MDR isolates highlighted in bold.

Sample	Category	[a]Subcategory (additional description, as provided by supplier)	[b]Lineage	Ampicillin	Chloramphenicol	Erythromycin	Gentamicin	Tetracycline
Clinical: total tested n=20								
CLM17	**Clinical**	**Unknown**	**I**	**-**	**R**	**R**	**R**	**R**
Environmental: total tested n=31								
MEN30	Environmental	Drain (preparation area)	I	-	R	R	-	-
MEN34	Environmental	Drain (production)	I	-	-	R	-	-
MEN26	Environmental	Drain (preparation area)	I	-	R	-	-	-
MEN31	Environmental	Drain (cooking area)	II	-	R	R	-	-
MEN47	Environmental	Drain	I	-	R	R	-	-
MEN01	Environmental	Drain (mixer)	I	-	-	-	R	-
MEN04	Environmental	Drain (mixer)	I	-	-	R	-	-
MEN48	Environmental	Equipment	I	-	-	R	-	-
MEN11	Environmental	Equipment	I	-	-	R	-	-
MEN36	Environmental	Floor	II	-	-	R	-	-
MEN38	**Environmental**	**Hand (during production)**	**II**	**-**	**R**	**R**	**-**	**R**
MEN14	Environmental	Surface (red cutting boards)	II	-	R	R	-	-
MEN15	Environmental	Surface (production tables)	II	-	-	R	-	-
MVA03	Environmental	Unknown	II	-	-	R	-	-

Continued on next page

Sample	Category	[a]Subcategory (additional description, as provided by supplier)	[b]Lineage	Ampicillin	Chloramphenicol	Erythromycin	Gentamicin	Tetracycline
Raw meats: total tested n=31								
MRA32	**Raw meats**	**Beef (mince)**	**II**	**-**	**-**	**R**	**R**	**R**
MRA32a	Raw meats	Beef (mince)	II	-	-	R	-	-
MRA18	**Raw meats**	**Beef**	**I**	**-**	**R**	**R**	**-**	**R**
MRA03	Raw meats	Beef (mince)	II	-	-	R	-	-
MRA31	Raw meats	Chicken (whole birds)	I	-	-	-	-	R
MRA34	Raw meats	Chicken (fresh breasts)	I	-	-	R	-	R
MRA42	Raw meats	Chicken (frozen thighs)	I	-	-	-	-	R
MRA43	Raw meats	Chicken (pieces)	II	-	-	-	-	R
MRA46	Raw meats	Chicken (mixed portions)	I	-	-	-	-	R
MRA17	Raw meats	Chicken (drumsticks)	I	-	-	R	-	R
MRA36	**Raw meats**	**Pork (BBQ portions)**	**II**	**-**	**R**	**R**	**-**	**R**
MRA23	Raw meats	Unknown	I	-	-	R	-	-
MRA26a	**Raw meats**	**Unknown**	**II**	**-**	**R**	**R**	**-**	**R**
MRA26	Raw meats	Unknown	II	-	R	R	-	-
MRA20	Raw meats	Unknown	I	-	R	R	-	-
MRA11	Raw meats	Unknown	I	-	-	R	-	-
MRA13	Raw meats	Unknown	I	-	-	-	R	-
MRA14	Raw meats	Unknown	I	-	-	R	-	-
MRA20a	Raw meats	Unknown	I	-	-	R	-	-
MRA21	Raw meats	Unknown	I	-	R	R	-	-
MRA33	Raw meats	Unknown	II	-	-	R	-	-
MRA30	Raw meats	Unknown	I	-	-	R	-	-

Continued on next page

Sample	Category	[a]Subcategory (additional description, as provided by supplier)	[b]Lineage	Ampicillin	Chloramphenicol	Erythromycin	Gentamicin	Tetracycline
Raw seafood: total tested n=61								
MRA02	Raw seafood	Seafood	I	-	-	-	-	R
MVA02	Raw seafood	Seafood	I	-	-	R	-	R
MVA05	Raw seafood	Seafood	I	-	-	R	-	-
MVA30	Raw seafood	Seafood	I	-	-	R	-	-
MVA38	Raw seafood	Seafood	I	-	R	-	-	-
MVA41	Raw seafood	Seafood	I	-	-	R	-	-
MVA44	Raw seafood	Seafood	I	-	-	R	-	-
MVA47	Raw seafood	Seafood	I	-	-	R	-	-
MVA56	Raw seafood	Seafood	I	-	-	R	-	-
MVA62	Raw seafood	Seafood	II	-	-	-	-	R
MVA65	Raw seafood	Seafood	I	-	-	R	-	-
Ready-to-eat: total tested n=34								
MRE21	Ready-to-eat	Deli meat (bacon breakfast griller)	II	-	-	R	-	-
MRE40	Ready-to-eat	Deli meat (cheese griller)	I	-	-	R	-	R
MRE17	Ready-to-eat	Deli meat (pepper beef)	II	-	-	R	-	-
MRA04	Ready-to-eat	Fresh produce	I	-	-	R	-	R
MRE32	**Ready-to-eat**	**Fresh produce (coriander after dipping)**	**I**	**-**	**-**	**R**	**R**	**R**
MRE38	Ready-to-eat	Hummus	II	-	R	-	-	-

Continued on next page

Sample	Category	aSubcategory (additional description, as provided by supplier)	bLineage	Ampicillin	Chloramphenicol	Erythromycin	Gentamicin	Tetracycline
MRE07	Ready-to-eat	Hummus	II	-	-	R	-	-
MRE08	Ready-to-eat	Hummus (red pepper)	II	-	-	R	-	-
Hb6	Ready-to-eat	Polony	II	-	R	-	-	-
Hb4	**Ready-to-eat**	**Polony**	**II**	**-**	**R**	**R**	**-**	**R**

[a]Isolates from the same category are not necessarily from the same retail outlet, or environmental origin. Isolates with similar descriptions are not referring to duplicates.

[b]These isolates were characterised into lineage groups by PCR-RFLPs [51].

AIMS Press

© 2021 the Author(s), licensee AIMS Press. This is an open access article distributed under the terms of the Creative Commons Attribution License (http://creativecommons.org/licenses/by/4.0)
